# Mycetoma Pulmonary Secondaries from a Gluteal Eumycetoma: An Unusual Presentation

**DOI:** 10.1371/journal.pntd.0004945

**Published:** 2016-10-06

**Authors:** Nancy Awad Mohamed, Ahmed Hassan Fahal

**Affiliations:** Mycetoma Research Centre, University of Khartoum, Khartoum, Sudan; Fundação Oswaldo Cruz, Brazil, BRAZIL

## Case Presentation

A 38-year-old teacher from the White Nile State in Sudan was referred to the Mycetoma Research Centre (MRC), University of Khartoum, Sudan, in 2009 with a massive gluteal eumycetoma. In 1996, he noted a small painless subcutaneous gluteal swelling, which gradually increased in size. Initially, a diagnosis of gluteal abscess was made, and he underwent drainage of a seropuruluent discharge at a district general hospital. This was followed by several wound debridements and regular dressing, but no diagnosis was established, and no medication was administered.

By 2003, the lesion involved a wide area of the gluteal region and the upper part of the right thigh. A diagnosis of eumycetoma was established by grain culture and histopathological examination of the surgical biopsy, and he was started on oral ketoconazole at 200 mg a day; however, the treatment was discontinued after three months because of the drug’s adverse reactions. These reactions included skin hyperpigmentation, lip dryness and fissuring, gynecomastia, and hepatic function disturbances.

**Fig 1:**
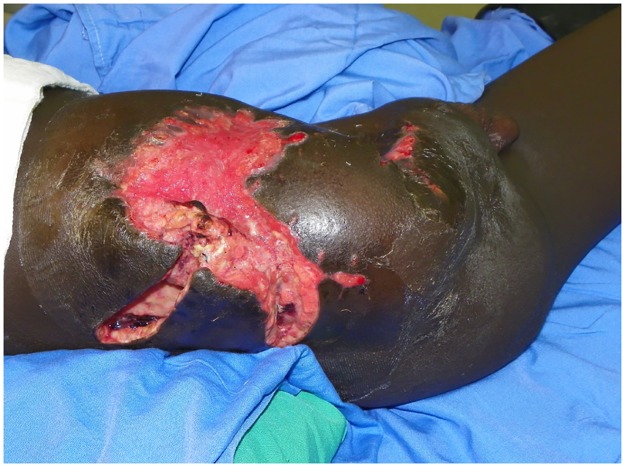
Photo showing massive disease involving the lower posterior and anterior abdominal walls, with massive skin affection, multiple sinuses, and disarticulation of the right lower limb.

He underwent multiple wide local surgical excisions, but the disease continued its aggressive course, and in 2005 he underwent right hip disarticulation for disease control. The disease recurred soon afterward in the lower back. In 2009, he was started on oral Ketoconazole at 800 mg a day and underwent several wide local excisions. The disease relentlessly progressed to involve the lower posterior and anterior abdominal walls (Fig 1). He was hospitalized several times to eradicate the concomitant bacterial infections and control the stump phantom pains with appropriate antibiotics and opiates.

**Fig 2:**
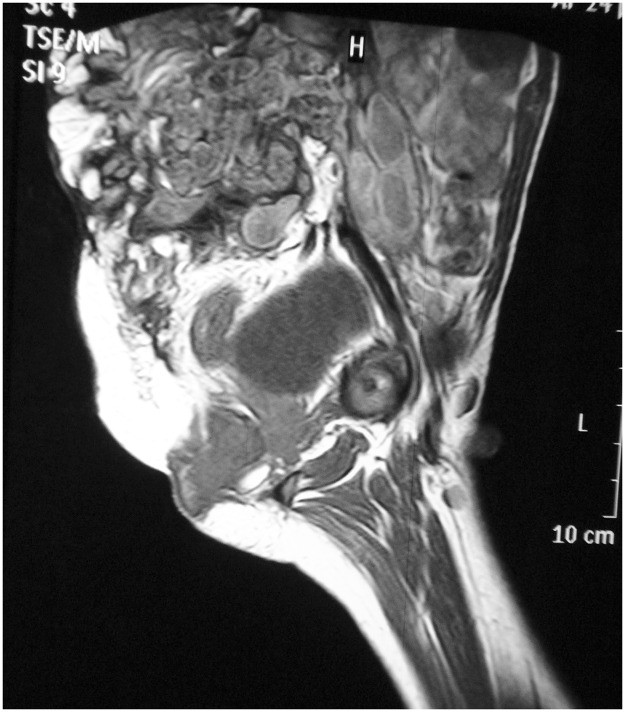
MRI showing an aggressive infiltrating lesion invading the pelvis and lumber region that extends from the posterior abdominal wall, with a sizable intrapelvic and intraabdominal extension infiltrating the lumber and sacral vertebrae and an intraspinal epidural extension displacing the abdominal organ to the left; in addition, there were many “dot-in sign” of mycetoma.

By 2012, he developed magnetic resonance imaging (MRI)-confirmed infiltration of the lumber and sacral vertebrae with intraspinal epidural extension (Fig 2), resulting in an inability to walk with his crutches, excruciating lower backache, difficulty in passing urine, and defecation. He was started on intravenous amphotericin B liposomal at 6 mg/kg on alternate days for four weeks with some improvement. This regime was followed by voriconazole at 800 mg a day for six months, but it had to be suspended because of abnormal hepatic function. Itraconazole at 800 mg a day was substituted.

**Fig 3:**
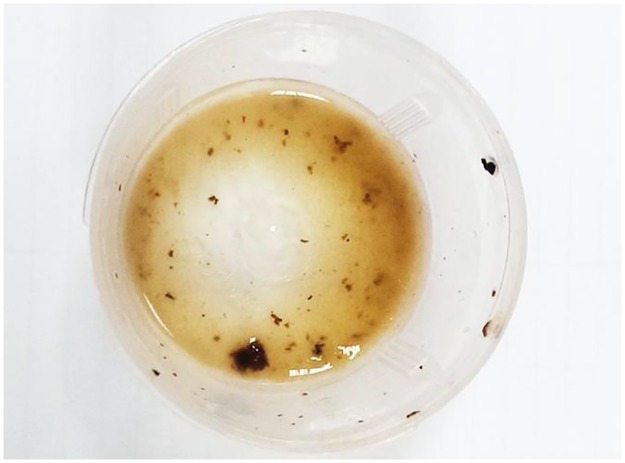
The macroscopic appearance of the purulent sputum with a small clump of mucoid substances and black grains.

In June 2015, he was admitted with severe watery diarrhea, dyspnea, and a productive cough containing black grains for a hospital stay of one month (Fig 3). On examination, he was unwell, emaciated, and in pain. He had finger clubbing and generalized edema. Respiratory examination revealed reduced chest movements with bulging of the left intercostal spaces, a dull percussion note, and decreased air entry over the left lung.

The abdominal examination revealed a distended abdomen, with numerous active sinuses in the lower anterior abdominal wall. There were large intra-abdominal masses, multiple active sinuses in the back with a huge mass involving the left flank.

A renal profile showed evidence of renal impairment, a hepatic profile revealed hypoalbuminemia, and a hematological profile revealed microcytic hypochromic anemia. Urinalysis showed pyuria. He was HIV negative.

**Fig 4:**
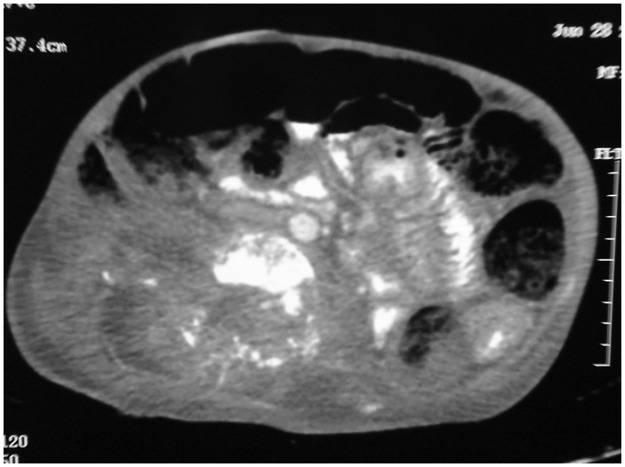
CT scan showing extensive cystic lesions and massive destruction of the lumber vertebrae, sacrum, and left iliac bone associated with paravertebral soft tissue mass extending to the psoas muscles.

**Fig 5:**
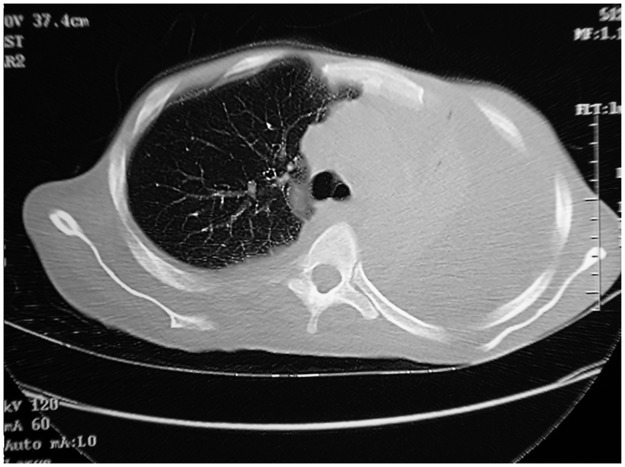
Chest CT scan showing a massive left pleural effusion with collapsed left lung.

An abdominal computed tomography (CT) scan revealed extensive cystic lesions and severe destruction of the lumber vertebrae, sacrum, and left iliac bone that was associated with paravertebral soft tissue mass extending to the psoas muscles (Fig 4). A chest CT scan showed a large left pleural effusion with collapsed lung (Fig 5). An intercostal chest drain was inserted, and several liters of seropurulent fluid with many floating black grains were drained.

The cytological examination of the sputum and pleural fluid showed numerous pus cells, lymphocytes, and histiocytes in a necrotic background. Several colonies of septate, branching fungal hyphae were embedded in a cement-like matrix indicative of *Madurella mycetomatis*. In addition, the pleural effusion showed *Candida albicans* pseudohyphae and yeast cells. The sputum culture revealed growth of *Escherichia coli* and *Pseudomonas aeruginosa*, which are sensitive to a wide range of antibiotics. Grains culture, PCR, and loop-mediated isothermal amplification (LAMP) molecular techniques confirmed the diagnosis of *M*. *mycetomatis* infection [1–3].

The immune status of the patient was determined. The levels of interferon gamma (IFN-), interleukin 12 (IL-12), tumor necrosis factor alpha (TNF-α), IL-10, IL-4, and IL-5 were measured in aliquots of cell-free supernatants by a sandwich ELISA using ELISA Max Deluxe Set (Catalog Number: 430104) after stimulation of whole blood with a culture filtrate of *M*. *mycetomatis*. The TNF-α, IL-10, and IL-5 levels were higher in the patient than in control subjects. The IFN-γ level was slightly increased in the patient compared to the control subjects. In contrast, the IL-12 level was lower than in the control subjects, whereas no production of IL-4 (0 pg/ml) was detected in the patient and healthy controls. The patient had severe CD4 lymphopenia at 104 CD4 cells/μl (normal range 450–1,350 cells/μl) with a total lymphocyte count of 2.6 x 103 lymphocytes/μl.

His hospital stay was stormy. He spent 15 days in the hospital, where he had continuous intravenous fluids and 3 units of blood and was on itraconazole (200 mg bd), cefuroxime (1.5 g per day), and Augmentin (1 g twice per day). His condition continued to deteriorate, resulting in severe sepsis, recurrent hypoglycemia, and multiorgan failure, and despite the intensive resuscitation, he sadly succumbed.

## Case Discussion

Mycetoma is an implantation mycosis, characterized by large tumor-like swellings, and it is commonly located in the extremities, involving the subcutaneous tissue and spreading locally to the skin, deep structures, and bones [4]. It can rarely spread to the regional lymph node and occasionally produces new satellites [5]. Blood spread in mycetoma is exceedingly uncommon [6]. It can be caused by taxonomically diverse microorganisms, both of bacterial (actinomycetoma) and fungal origin (eumycetoma) [7]. The disease is mainly found in tropical and subtropical regions of the world, and the majority of patients are reported from Mexico, Senegal, Sudan, and India, but its true prevalence and incidence are not well defined [8]. Furthermore, there are no rapid diagnostic tools, and its treatment, especially for eumycetoma, is unsatisfactory, resulting in high morbidity, including amputation of limbs (“see Key Learning Points”).

It has been suggested that continuous intestinal peristalsis prevents the inoculation of mycetoma and, hence, abdominal cavity involvement is rare in mycetoma [9]. However, in our reported patient, the mycetoma indeed found its way to the pelvic and abdominal cavities. The lungs in our patient were involved, and this pulmonary spread of mycetoma has not been previously reported. We postulated that this was because of spread from the abdominal cavity or hematological spread The presence of *C*. *albicans* pseudohyphae and yeast cells in the pleural fluid is likely due to the prolonged use of antibiotics. The poor response to the various therapeutic modalities in our patient is multifactorial because of depressed immunity, recurrent superimposed bacterial infection, massive disease burden, and the resistance of the causative organism to antifungal therapy.

The high production of the cytokines IL-10 and IL-5 with CD4 lymphopenia is likely to be a cause of the aggressive disease and the pulmonary spread.

Mycetoma was only recently listed by the World Health Organization (WHO) as a neglected tropical disease (NTD). Perhaps this important step will facilitate research to produce rapid diagnostic tools and effective and safe therapeutic options.

### Ethics Statement

Written informed consent was obtained from the patient.

**Key Learning Points**

- Mycetoma is a localized disease but rarely can spread by the lymphatics and blood.
- Mycetoma can have a progressive, aggressive, and wild clinical course.
- Mycetoma can spread to distant organs such as the lung and spinal cord.
- Some patients may not respond to the available medical and surgical treatment, and this can result in fatality.
- The depressed immune system in the patient described here may be the cause of the aggressive disease and pulmonary spread.
